# Observation of the Charge Density Wave Excitonic Order Parameter in Topological Insulator Monolayer WTe_2_

**DOI:** 10.1021/acsnano.5c08005

**Published:** 2025-09-04

**Authors:** Liam Watson, Joan Ripoll, Zhengjue Tong, Amit Kumar, Yande Que, Yang-Hao Chan, Hsin Lin, Shantanu Mukherjee, Manuela Garnica, Mark T. Edmonds, Michał Papaj, Amadeo L. Vazquez de Parga, Bent Weber, Iolanda Di Bernardo, Michael S. Fuhrer

**Affiliations:** † School of Physics and Astronomy, 2541 Monash University, Clayton, VIC 3800, Australia; ‡ Australian Research Council Centre of Excellence in Future Low-Energy Electronics Technologies, Monash University, Clayton, VIC 3800, Australia; § 202527 Instituto Madrileño de Estudios Avanzados en Nanociencia (IMDEA-Nanociencia), Madrid 28049, Spain; ∥ Departamento de Física de la Materia Condensada, 16722 Universidad Autónoma de Madrid, Madrid 28049, Spain; ⊥ School of Physical and Mathematical Sciences, 54761 Nanyang Technological University, Singapore 637371, Singapore; # Institute of Atomic and Molecular Sciences, 38017 Academia Sinica, Taipei 106319, Taiwan; ∇ Institute of Physics, Academia Sinica, Taipei 155201, Taiwan; ○ Quantum Centre for Diamond and Emergent Materials, 37268 Indian Institute of Technology Madras, Chennai 600036, Tamil Nadu, India; ◆ Instituto Nicolás Cabrera, Universidad Autónoma de Madrid, 28049 Madrid, Spain; ¶ Department of Physics, 14743 University of Houston, Houston, Texas 77204, United States; ⋈ Departamento de Física de la Materia Condensada, Universidad Autónoma de Madrid, Madrid 28049, Spain; ⧓ IFIMAC Condensed Matter Physics Center, Madrid 28049, Spain; 13 Department of Physics, 37268 Indian Institute of Technology Madras, Chennai 600036, Tamil Nadu, India; 14 Center for Atomistic Modelling and Materials Design, 37268 Indian Institute of Technology Madras, Chennai 600036, Tamil Nadu, India

**Keywords:** excitonic order parameter, charge density wave, topological excitonic insulator, edge state, monolayer tungsten ditelluride (WTe_2_)

## Abstract

Strong electron–hole interactions in a semimetal or narrow-gap semiconductor may drive a ground state of condensed excitons. Monolayer WTe_2_ has been proposed as a host material for such an exciton condensate, but the order parameter—the key signature of a macroscopic quantum-coherent condensate—has not been observed. Here, we use Fourier-transform scanning tunneling spectroscopy (FT-STS) to study quasiparticle interference (QPI) and periodic modulations of the local density of states (LDOS) in monolayer WTe_2_. In WTe_2_ on graphene, in which the carrier density can be varied via back-gating, FT-STS shows QPI features in the two-dimensional (2D) bulk bands, confirming the interacting nature of the bandgap in neutral WTe_2_ and the semimetallic nature of highly n- and p-doped WTe_2_. We observe additional nondispersive spatial modulations in the LDOS imprinted on the topological edge mode of neutral WTe_2_ on metallic substrates (graphene and graphite), which we interpret as the interaction of the topological edge mode with the expected charge density wave order parameter of the excitonic condensate in WTe_2_ at low interaction strength due to screening by the metallic substrates.

## Graphical abstract

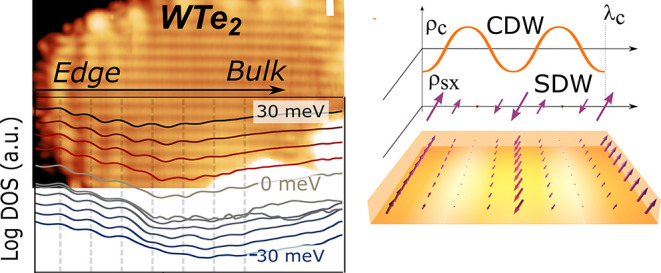

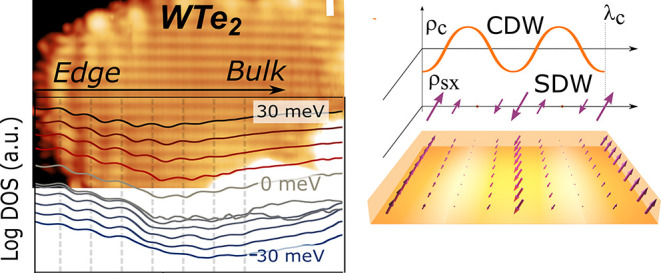

## Introduction

In a semimetal or narrow-gap semiconductor, the Coulomb interaction can drive a unique many-body ground state in which excitons, composite bosons of strongly bound electrons and holes, condense. The exciton condensate is a macroscopic quantum-coherent state, analogous to a superconductor, with a Bardeen–Cooper–Schrieffer (BCS)-like order parameter.^1–4^ However, because excitons are charge-neutral, the condensate allows no supercurrent and is an example of an excitonic insulator. The existence and behavior of the exciton condensate is highly conditional on the electron and hole densities and the strength of the Coulomb interaction (*U*), and so is expected to depend strongly on doping, electric fields, and screening from the dielectric environment (where *U* ∝ ε^–1^; ε is the dielectric constant). Monolayers of WTe_2_ are theoretically predicted to host an exciton condensate with at least three possible phases (illustrated in Figure 1) which will be referred to as SDW/CDW, spin spiral, and T‐CDW respectively. In the time-reversal symmetry breaking SDW/CDW phase at low *U*, the order parameter exhibits a spin density wave (SDW) with wavevector *q*_c_ and a charge density wave (CDW) with wavevector 2*q*_c_,^5,6^ where *q*_c_ is expected to be roughly equal to the separation of the electron and hole pockets in momentum space. At high values of *U*, however, the condensate is expected to exhibit a time-reversal breaking spin spiral phase, which is absent of both charge and spin density modulations, but these can be weakly revealed upon application of a magnetic field.^6,7^ A time-reversal symmetry preserving phase T‐CDW has been described, which is nearly degenerate in energy to the spin spiral phase, and exhibits a CDW order parameter, but with half the wavevector (*q*_c_) and is absent of any spin order.^5,8^

**Figure 1:**
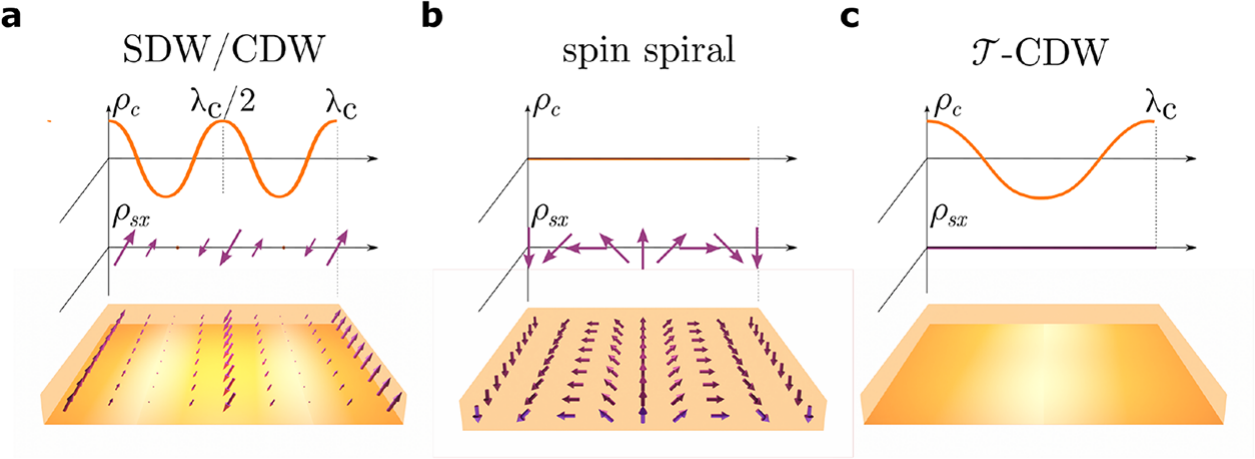
Schematic of the excitonic phases in WTe_2_. The real-space orders are defined in terms of wavelength λ_c_ = 1/*q*_c_. (a) SDW/CDW phase, exhibiting a charge density (ρ_c_) with a period λ_c_/2 and spin density (ρ_s*x*_) with a period λ_c_. (b) Spin spiral phase—lacking both charge and spin density—exhibits a spin rotation about the *y*-axis with period λ_c_. (c) T‐CDW phase, exhibiting charge order with period λ_c_ and lacks spin order.

Recent experimental evidence^7,9,10^ suggests monolayer WTe_2_ to be a topological excitonic insulator (EI), with conducting topological edge modes and an insulating bulk which is insensitive to charge addition^7,10^ up to a critical threshold at which the gap collapses.^9^ However, direct evidence for an exciton condensate in WTe_2_, e.g., the existence of a symmetry-breaking order parameter indicating spontaneous macroscopic coherence, is lacking. The failure to observe the order parameter in the WTe_2_ ground state is not understood, but it has been speculated to be due to the difficulty of detecting the spin spiral phase with no charge ordering, which is expected in some experimental circumstances.^6,10^ CDW-like oscillations have been observed in early reports of monolayer WTe_2_,^11,12^ but those samples displayed a semimetal-like local density of states (LDOS) with a Coulomb gap (CG) centered at the Fermi energy. Fourier transform scanning tunneling spectroscopy (FT-STS) spectra on those samples revealed quasiparticle interference (QPI) features from overlapping electron and hole pockets, and so the possibility of charge order was rejected in favor of interpocket scattering around the Fermi level.^12^ Recently, evidence of edge LDOS modulations of order *q*_c_ were observed in samples of monolayer WTe_2_ on graphene,^9,13^ although a systematic analysis of their dispersion was not undertaken.

Here, we investigate the electronic structure of the monolayer WTe_2_ using FT-STS. The topological nature of WTe_2_ is confirmed via the observed gapless edge mode. We resolve QPI features characteristic of the insulating bulk band structure, with a single valence band maximum at zero wavevector Γ and two conduction band minima at wavevector Λ ≈ *q*_c_, separated by an energy gap. Charge injection via gating of the WTe_2_ causes the energy gap to close at critical electron and hole densities, whereupon QPI directly reveals that the resulting gapless states exhibit semimetallic behavior. The QPI features are in good agreement with the calculated interacting *k*·*p* model band structure. Near the sample edges, we observe similar QPI features but also additional modulations of the LDOS which decay from the edge into the bulk, that are nondispersive, persist through the bandgap, and do not originate from QPI. We interpret these LDOS modulations as the signature of the charge density wave exciton condensate order parameter at 2*q*_c_, imprinted on the topological edge state, confirming the quantum-coherent nature of the insulating state.

## Results

Figure 2 shows a summary of our experimental evidence for an exciton condensate order parameter in WTe_2_. Monolayer WTe_2_ was grown via molecular beam epitaxy (MBE) on two different sample architectures shown in Figure 2a,b: exfoliated graphene on 300 nm SiO_2_ over p-type silicon and highly oriented pyrolytic graphite (HOPG), respectively. Graphite and graphene were chosen to ensure negligible substrate coupling effects^14^ and high conductivity at ultralow temperatures. The carrier concentration in the graphene was tuned by application of a voltage *V*_G_ to the p-type silicon back-gate. Charge transfer between the graphene and epitaxial WTe_2_ is driven by the generated chemical potential offset, facilitating electrostatic doping.^9^ The crystallization in a rectangular 1T′ lattice (*a* = 3.48 ± 0.03 Å and *b* = 6.17 ± 0.07 Å) was confirmed by low-energy electron diffraction (not shown) and atomically resolved scanning tunneling microscopy (STM) images (Figures 2c and S2).

**Figure 2:**
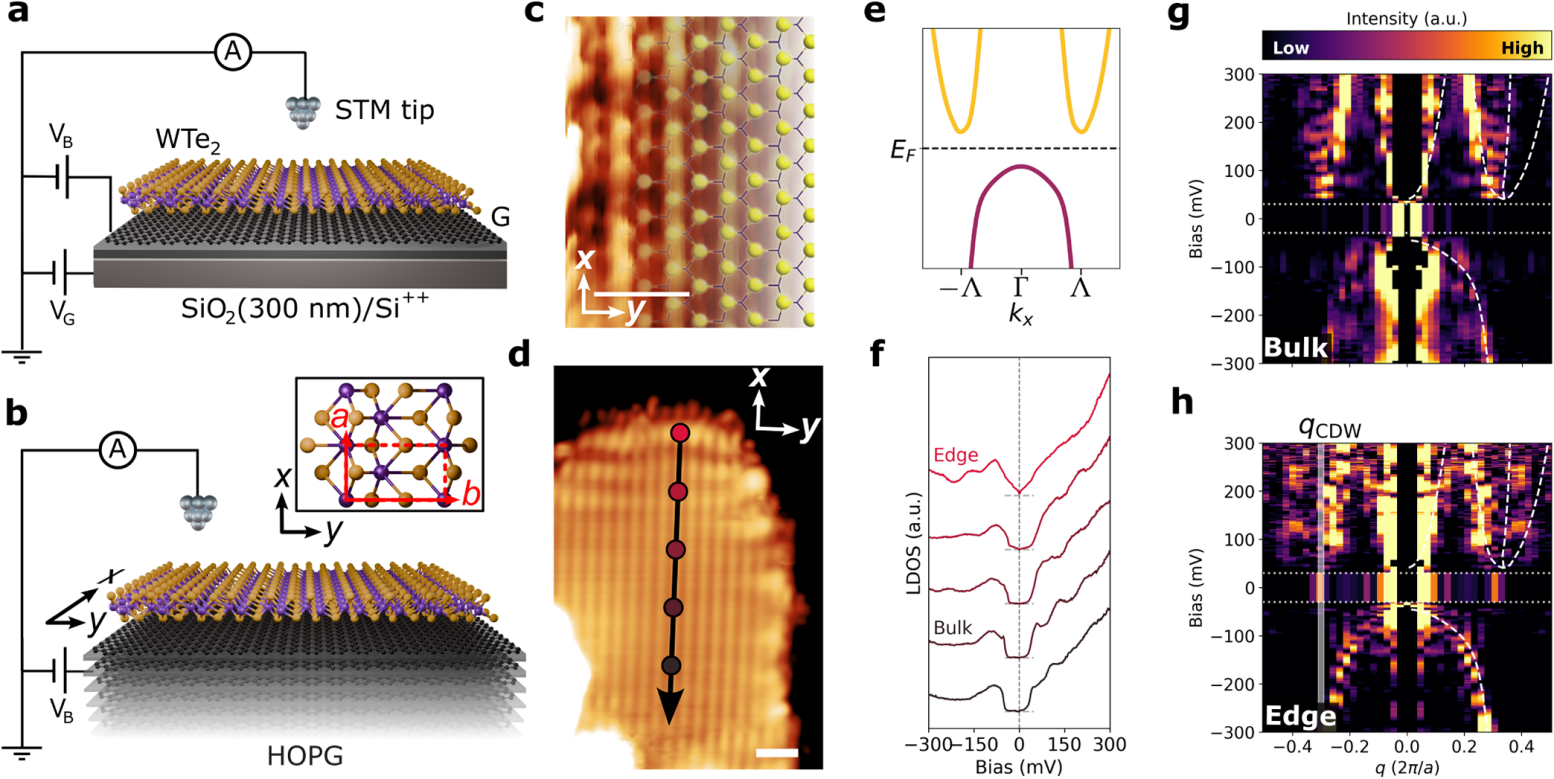
Experimental overview. (a, b) Schematic of STM experimental setups. Epitaxial submonolayer WTe_2_ grown on (a) graphene (G) over SiO_2_/Si, and (b) HOPG. The inset of panel (b) shows the top-down view of the WTe_2_ lattice; unit cell and lattice vectors are indicated. Sample (*V*_B_) and back-gate (*V*_G_) electrode biases are indicated. (c) Topography of the monolayer WTe_2_ surface grown on HOPG, overlaid with the terminating Te atoms (yellow balls). Scale bar: 1 nm. (d) Topography of WTe_2_ on HOPG. Lattice directions are indicated. Scale bar: 2 nm. (e) Interacting *k*·*p* band structure along the *k*_*x*_ direction. The Fermi energy (*E*_F_), Brillouin zone center (Γ) and conduction band minima (±Λ) are indicated. (f) LDOS measured along the black arrow in panel (d) at the points indicated. (g, h) FT-STS spectra for (g) bulk, and (h) edge regions of WTe_2_, acquired from the architectures in (a) and (b), respectively. The gap energies (between −30 and +30 meV) are integrated for the sake of clarity. The calculated scattering vectors from the interacting *k*·*p* band structure and the measured CDW wavevector are indicated with white dashed lines and the white shaded region, respectively. STM/STS parameters: (c, d) *V* = 50 mV, *I* = 100 pA, (g) *V*_set_ = 500 mV, *I*_set_ = 2 nA, *V*_mod_ = 5 mV, *f*_mod_ = 726 Hz, 256 spectra over 16.6 nm, indicated by the arrow in Figure S3a. (f, h) *V*_set_ = 300 mV, *I*_set_ = 1 nA, *V*_mod_ = 2.3 mV, *f*_mod_ = 736 Hz, 128 spectra over 15.0 nm, indicated by the arrow in panel (d).

Scanning tunneling spectroscopy (STS), which measures the energy dependent LDOS, was performed at points along a line from the monolayer edge into the bulk along the negative *x*-direction indicated in Figure 2d, which corresponds to the cut along *k*_*x*_ in the Brillouin zone, intersecting both the hole pocket (centered at Γ) and electron pockets (centered at ±Λ) (Figure 2e). Figure 2f shows that the insulating interior is confirmed in STS via the observation of a hard gap of width ∼60 meV roughly centered around the Fermi energy, while the presence of an enhanced, gapless LDOS at the edge of the island provides direct evidence of the monolayer’s topological character, i.e., the existence of a topological edge state as predicted by the bulk-boundary correspondence.

Figure 2g,h summarizes our FT-STS observations in the insulating interior and near the edge of the WTe_2_ island, where topological edge states are observed, respectively. These figures are repeated in Figures 3b and 4e, respectively, in which they are discussed in greater detail. Figure 2g shows the FT-STS spectrum along *k*_*x*_ in the insulating interior, which reveals the expected dispersing QPI features of the electron and hole pockets, separated by the bandgap. The electron pocket shows minima at *q* ≈ ±2Λ, reflecting interpocket scattering at approximately twice the wavevector *q* = 2*k*. Intrapocket scattering of the electron pockets is also seen at low *q*. The single valence band at Γ shows only intrapocket scattering at *q* = 2*k* as expected. These are confirmed by the overlaid calculated scattering vectors from the interacting *k*·*p* band structure (white dashed lines, see Experimental Section). An FT-STS spectrum acquired along the line in Figure 2d that includes the topological edge state (Figure 2h) displays similar dispersing QPI features. Interestingly, additional modulations of the LDOS are observed within the gap at wavevector *q*_CDW_ = 0.29 ± 0.01­[2π/*a*], which are nondispersive. The existence of nondispersive LDOS modulations within the gap is inconsistent with QPI as an origin, as there are no bulk bands to contribute scattering wavevectors, and the topological edge mode parallel to *x* is strongly dispersive within the gap, connecting the valence and conduction bands across the Brillouin zone boundaries along *k*_*x*_, not across Γ.^15^ We instead interpret these LDOS modulations as the signature of the CDW order parameter of the exciton condensate, imprinted on the topological edge state, and we explore these features in more detail.

**Figure 3:**
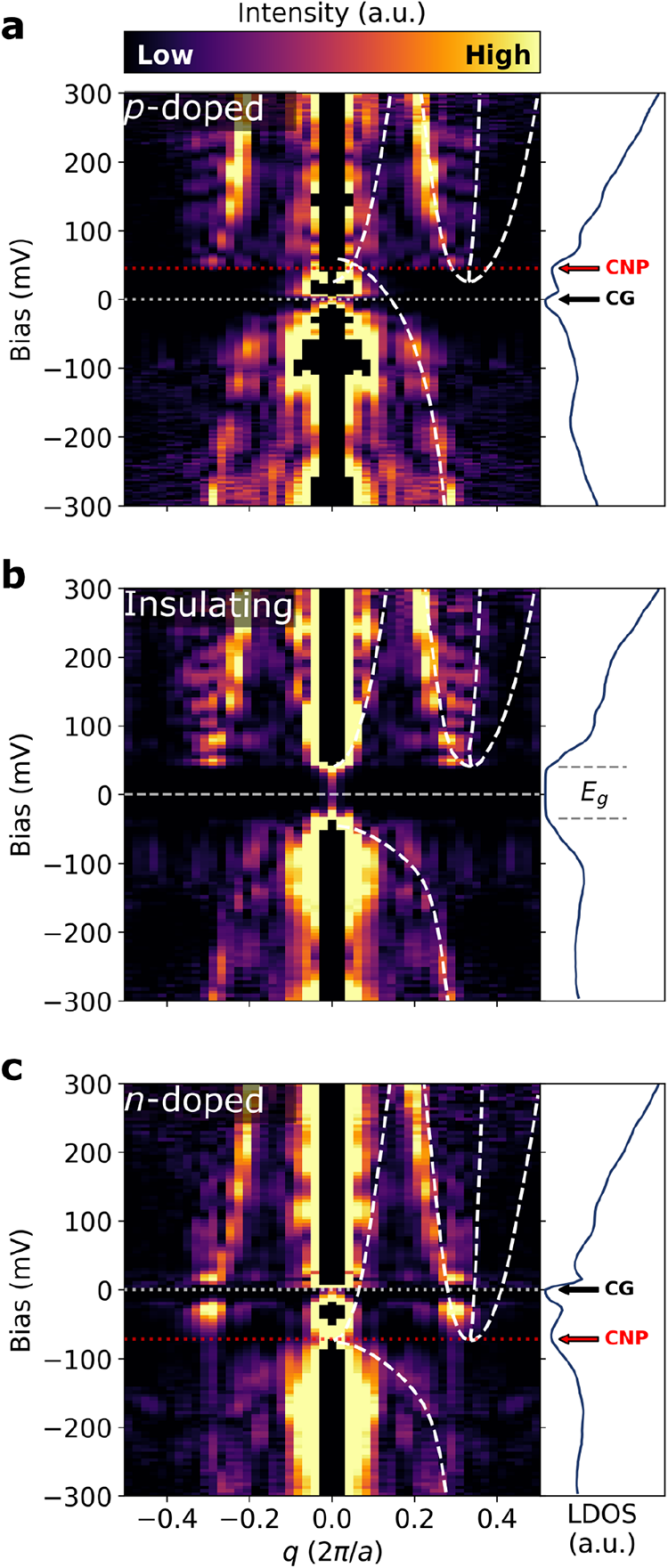
FT-STS spectra from the monolayer WTe_2_ two-dimensional (2D) bulk (i.e., excluding island edges) grown on graphene over SiO_2_/Si for various back-gate potentials: (a) p-doped (*V*_G_ = −40 V), (b) insulating (*V*_G_ = 10 V), and (c) n-doped (*V*_G_ = +70 V). The calculated scattering vectors from the interacting *k*·*p* band structure are indicated with white dashed lines. Each FT-STS spectrum is accompanied by a corresponding integrated LDOS spectrum. The bandgap (*E*_g_), charge neutrality point (CNP) and Coulomb gap (CG) are indicated. STS parameters: *V*_set_ = 500 mV, *I*_set_ = 2 nA, *V*_mod_ = 5 mV, and *f*_mod_ = 726 Hz.

**Figure 4:**
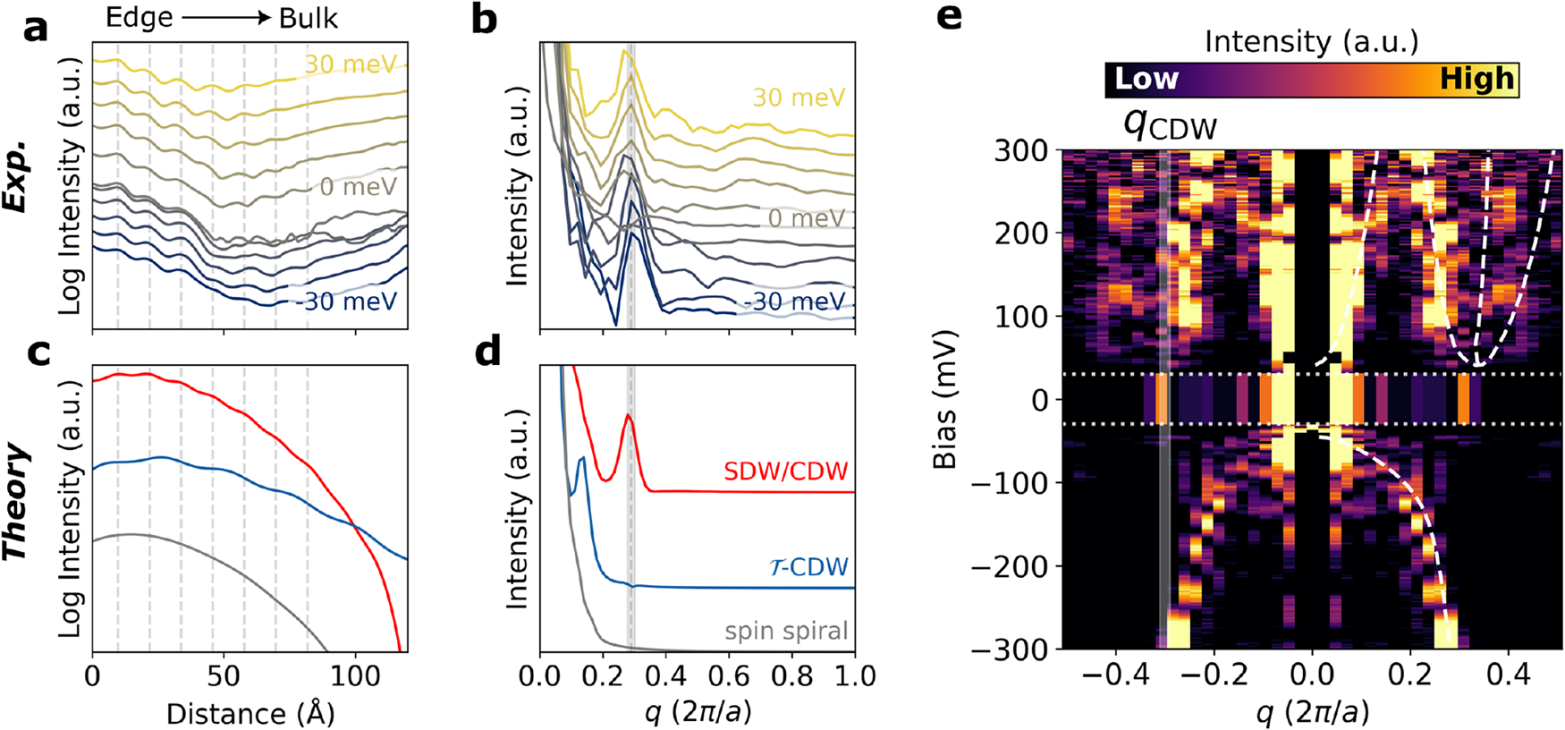
LDOS oscillations coupled to the topological edge state of monolayer WTe_2_ grown on a HOPG. (a) Constant energy cuts of the LDOS inside the insulating gap as a function of distance away from the edge, acquired at *B*_⊥_ = 3 T. (b) Fourier transform of (a). (c) Calculated topological edge LDOS and (d) Fourier transform for the three different excitonic phases: SDW/CDW (red), T‐CDW (blue), and spin spiral (gray), offset for clarity. The approximate oscillation period in real space is indicated with vertical dashed lines in parts (a) and (c). The mean peak frequency and resolution in Fourier space are indicated by the gray dashed line and shaded region in parts (b) and (d). (e) FT-STS spectrum of the edge region of monolayer WTe_2_. The gap energies (between −30 and +30 meV) are integrated for clarity. The calculated scattering vectors from the interacting *k*·*p* band structure and the measured CDW wavevector are indicated with white dashed lines and the white shaded region, respectively. STS parameters: *I*_set_ = 1 nA, *f*_mod_ = 736 Hz, (a, b) *V*_set_ = 100 mV, *V*_mod_ = 0.7 mV, (e) *V*_set_ = 300 mV, *V*_mod_ = 2.3 mV.

First, we use QPI features in FT-STS to explore the nature of the gap-collapse transition upon doping and confirm the origin of the bandgap in the insulating state, where strong Coulomb interactions renormalize the band dispersion. Figure 3 shows the FT-STS spectra in the insulating interior over different conditions of charge transfer doping. The insulating state (at low *V*_G_ = 10 V, Figure 3b, identical to Figure 2g) shows the expected band dispersion in the bulk, with the valence and conduction bands well separated by an insulating gap of approximately 60 meV. As previously shown,^9^ significant doping with holes or electrons induces a quantum phase transition, indicated by the collapse of the insulating gap. Here, we provide additional clear evidence of this collapse from QPI features in our gated FT-STS experiments. In p-doped WTe_2_ at *V*_G_ = −40 V (Figure 3a), the band edges appear to overlap at the charge neutrality point (CNP), indicated by the local LDOS minimum. A small Coulomb gap (CG) remains centered at the Fermi energy consistent with early reports of as-grown semimetallic WTe_2_.^11,12^ The CNP and CG are reflected in the accompanying integrated LDOS spectrum. A similar effect is observed for significant electron doping (*V*_G_ = +70 V, Figure 3c), inducing an n-doped semimetallic band structure. QPI confirms that the n- and p-doped states are both semimetallic, via the clear observation of the coexistence of electron and hole pockets near the Fermi energy in Figure 3a,c. This is reflected in the integrated LDOS, where the excitonic gap collapses to the small CG and the CNP aligns with the meeting of the bands. The calculated scattering vectors from the interacting *k*·*p* band structure align very well with the band positions. Nondispersing LDOS modulation at *q*_CDW_ was not observed in these spectra, acquired in the insulating 2D bulk.

Figure 4 examines the FT-STS of WTe_2_ near the topological edge of the sample in more detail. Figure 4a,b shows the real-space (Figure 4a) and Fourier-space (Figure 4b) variation of LDOS at various energies within the bulk gap (between −30 and +30 meV), taken along the line shown in Figure 2d, and acquired at a perpendicular magnetic field of strength 3 T. The results shown in Figures S4 and S5 indicate that the same physics is described at all field strengths (*B*_⊥_ = 0, ±3 T). The resolution of the CDW Fourier peaks is primarily limited to the decay length of the edge state in the bulk. In addition to the exponential decay of the edge LDOS into the interior expected for the topological edge mode, we observe LDOS oscillations, which appear to be modulations of the topological edge-derived LDOS (their roughly constant amplitude on the vertical logarithmic scale indicates the oscillation amplitude is proportional to the exponentially decaying LDOS of the topological edge). Figure 4b shows, remarkably, that the modulations of the LDOS are nondispersive, with a mean frequency of *q*_CDW_ = 0.29 ± 0.01­[2π/*a*]. Figure 4c,d features complementary theoretical calculations of the edge LDOS (at *E* = 3.5 mV) and corresponding Fourier transform respectively for the three excitonic phases (CDW/SDW, T‐CDW , and spin spiral). The edge LDOS shows good qualitative agreement with the CDW/SDW phase, and the measured wavevector *q*_CDW_ is consistent with the expected SDW/CDW order parameter of 2*q*_c_ ∼ 0.31­[2π/*a*].^6,16,17^ These features cannot be a result of QPI or Friedel oscillations as both these phenomena rely on the scattering of charges across constant energy contours in the band structure.^18,19^ Particularly, the latter is dispersive^20,21^ and only observed for systems with metallic Fermi surfaces^22^ which fundamentally conflicts with the insulating state observed in the interior.

Figure 4e (identical to Figure 2h) shows the FT-STS spectrum for the same line shown in Figure 2d, over a wider range of energies outside the gap. Similar to Figure 3b, the electron and hole pocket intrapocket scattering vectors are well resolved; however, the electron pocket interpocket scattering is less visible in this measurement. These features align well with those calculated from the interacting *k*·*p* band structure (see Supporting Information for details). Additional nondispersive features at *q* ≃ ±0.29­[2π/*a*] are observed inside the integrated in-gap spectrum, matching those seen in Figure 4b. Whether the nondispersive feature persists at positive energies is difficult to determine due to the strong interpocket scattering features at *q* ≈ 2Λ associated with the electron pockets.

## Discussion

The measured CDW order parameter 2*q*_c_ = 0.29 ± 0.01­[2π/*a*] aligns well with experimentally derived values of Λ from angle-resolved photoelectron spectroscopy (ARPES) measurements, namely 2Λ ≈ 0.3 ^16^ and 0.33­[2π/*a*].^17^ The overlaid calculated scattering vectors from the interacting *k*·*p* band structure appear to fit the dispersing QPI features of the electron and hole pockets well in the FT-STS spectra.

Naively, the CDW order parameter expected in the general case using a two-band model^3^ is simply *q*_c_ = Λ, equal to the separation between the electron and hole pockets in momentum space. The excitonic ground state T‐CDW with order parameter of *q*_c_ = Λ is predicted for WTe_2_ when time-reversal symmetry preservation is enforced,^5^ but it is found to have slightly higher energy than its symmetry-breaking counterparts (SDW/CDW and spin spiral states).^6^ Calculations of the T‐CDW state^5^ predict modulations of the edge LDOS with period 1/Λ, producing nondispersive features at *q* = ±Λ inside the bandgap. We detect no strong features at *q* = ±Λ in both the edge and interior regions, and the LDOS modulations visible in real space exhibit a period clearly smaller than 1/Λ.

The topological properties of WTe_2_ are expected to be sensitive to time-reversal breaking phenomena, potentially opening a gap in the edge state.^8^ While the SDW/CDW and spin spiral phases locally break time-reversal symmetry, integrating over the period of the SDW and spiral (1/Λ ∼ 2 nm) results in no net magnetic moment.^5^ We do not observe a clear signature of a gap at the edge, as reflected in the intensity of the edge state LDOS modulations (Figure 4a), which are primarily independent of energy. Even in the presence of a ±3 T out-of-plane magnetic field (see Figures S4 and S5), persistence of enhanced edge LDOS within the bandgap energies is observed, which is consistent with transport measurements^23^ in which edge conduction persists. The culmination of this evidence strongly indicates that the topological edge state persists in the presence of an excitonic condensate in the interior, which coincides with theoretical predictions^5,8^ for the SDW/CDW state. Interaction of the SDW/CDW and spin spiral state orders with the edge state can potentially open small energy gaps at the folded Brillouin zone boundaries, i.e., at *k* = ±*q*_c_/2. We do not observe any suppression of LDOS larger than the Tomonaga–Luttinger liquid pseudogap^13^ due to formation of minigaps, nor any indication of a smeared or suppressed feature around the gap. This is possibly influenced by a combination of limited energy resolution and local disorder in the samples.

The phase diagram of the two time-reversal symmetry-breaking excitonic phases^6,24^ predicts that the SDW/CDW phase occurs at a much smaller magnitude and range of Coulomb interaction strengths compared to the spin spiral phase. Our work utilizes the conducting substrates graphene and graphite, expected to provide strong screening and reduced Coulomb interaction. The experiments in Figure 4 use a graphite substrate; at low frequency and long wavelength graphite’s screening is metallic, but at energies of 0.5–1.0 eV interband screening in graphite produces dielectric behavior with ε in the ranges of 14–40,^25^ which is in reasonable agreement with the range of substrate dielectric constants (17–39) expected to produce the CDW/SDW regime in.^6,24^ However, quantitatively determining the Coulomb interaction screening in experimental devices is nontrivial, and therefore we identify the EI phase based on the fact that it exhibits a particular periodicity of charge oscillations. Transport experiments^10^ on WTe_2_ encapsulated by hexagonal boron nitride (h-BN) have been assumed to be in the spin spiral phase due to the low dielectric constant of h-BN (ε_h‑BN_ = 3.5 ^26^). This raises the possibility that transport experiments may not study the same phase as our (and other) spectroscopic measurements. To our knowledge, all spectroscopic (ARPES, STS) studies of monolayer WTe_2_ have been performed on conducting substrates, with the exception of Jia et al., who performed STS and planar tunneling measurements on WTe_2_ encapsulated with h-BN. Interestingly Jia et al. may have observed a substantially larger bandgap, up to 109 meV, in STS of h-BN encapsulated WTe_2_, potentially due to a different phase of the exciton condensate,^24^ or significant strain^27,28^ from exfoliation and transfer techniques involved in the fabrication such devices.

The question remains as to why the CDW order parameter is not observed uniformly everywhere in the WTe_2_ interior. At positive energies, the CDW wavevector is expected to overlap with the electron pocket interpocket scattering QPI feature located at *q* ≈ 2Λ, making unambiguous observation of the CDW difficult at positive bias. At negative energies, a QPI feature due to interpocket scattering at *q* ≈ 2Λ is not expected nor observed; however, a nondispersive feature due to the CDW is also absent. The simplest explanation is that the effect of CDW on the bulk LDOS is simply too weak to be observed. The conducting topological edge state provides LDOS around the Fermi energy which can respond to the CDW potential providing a visible signature,^5^ in contrast to the insulating bulk. As shown in Figures 4a and S5, the LDOS oscillations persist for the entire decay length and decay in intensity at the same rate as that of the edge state. Therefore, it is unlikely that the LDOS oscillations originate from edge-specific phenomena such as local strain, lattice reconstruction, charge redistribution, or hybridization with substrate states, which are expected to dissipate on a shorter length scale. We note that a nondispersive feature at *q* = ±1/4­[2π/*a*] which overlaps with the hole pocket at negative energies is sometimes observed in the unfiltered data (see Figure S6), possibly indicating that this is an edge effect. Alternatively, the double derivative of these spectra (Figure S7) seems to indicate that this feature is dispersive and could provide evidence of band folding.

## Conclusions

In summary, we have observed the symmetry-breaking order parameter of the exciton condensate ground state in monolayer WTe_2_. The wavevector of the order parameter (*q* = 0.29 ± 0.01­[2π/*a*]) and the absence of gap opening at the edge (absence of strong time-reversal symmetry breaking) indicate that the order parameter is the predicted SDW/CDW order occurring at relatively weak interaction strength,^6,24^ consistent with strong screening provided by the metallic (HOPG and graphene) substrates. We find that the visualization of the order parameter in tunneling experiments is intimately tied to the topological edge LDOS, in line with recent theoretical predictions.^5^ This could provide a reason as to why it has not been captured until now, especially for studies utilizing exfoliated monolayer WTe_2_ samples.

## Experimental Section

WTe_2_ submonolayer crystals were synthesized via molecular beam epitaxy (MBE). Two different sample architectures were employed: exfoliated (monolayer) graphene on 300 nm SiO_2_ over p-type silicon and HOPG, each grown in their respective system.

### MBE on Graphene over SiO_2_/Si

Samples were prepared in an Omicron Lab10 ultrahigh vacuum (UHV) MBE chamber^13^ (base pressure <1 × 10^–10^ mbar). Fabrication of the graphene/SiO_2_/Si devices was completed in UHV and inert Ar atmosphere to facilitate clean and uncontaminated surfaces (see ref 9 for details). MBE growth of WTe_2_ crystals was carried out on graphene/SiO_2_/Si substrates held at 160 °C using codeposition of W (99.998%) and Te (99.999%) with a flux ratio of 1:280 for 1 h to achieve ∼40–50% monolayer coverage. W and Te atoms were evaporated using an e-beam evaporator (Focus GmbH) and a valved cracker cell (Createc GmbH), respectively. The fluxes of W and Te, monitored by a beam flux monitor (Dr. Eberl MBE-Komponenten GmbH), were optimized to 0.5 × 10^–10^ and 1.4 × 10^–8^ mbar, respectively.

### MBE on HOPG

HOPG bulk crystals (SPI supplies—grade A) were cleaved under nitrogen gas flow before being transferred to UHV (base pressure <2.7 × 10^–10^ mbar). The graphite substrates were degassed at 150 °C. While maintaining this substrate temperature, MBE growth of WTe_2_ was carried out via e-beam evaporation (Focus GmbH) of W (99.998%, flux = 10–11 nA) and effusion (Kentax GmbH, dual cell) of Te (99.999%, *T* = 340 °C) for 30 min to achieve ∼90% monolayer coverage.

### STM/STS

Samples grown on graphene over SiO_2_/Si were measured in a Unisoku mK-USM1600 low-temperature STM^29^ (base temperature ∼30 mK, junction temperature ∼150 mK, base pressure <1 × 10^–10^ mbar), while those grown on HOPG were measured in a SPECS Joule–Thomson low-temperature STM (base temperature ∼1.1 K, base pressure <1 × 10^–10^ mbar). Both STM systems utilized chemically etched W or mechanically cut Pt/Ir tips, which were calibrated using the Au(111) Shockley surface state before spectroscopic measurements. Standard lock-in techniques were utilized for spectroscopic measurements. Modulation amplitudes and frequencies can be found in the accompanying figure descriptions.

### Fourier Transform STS Data Processing

Fourier transform STS spectra were obtained by taking the Fourier transform of LDOS spectra acquired along the *x* (short)-axis of WTe_2_. The LDOS spectra were first treated with a Hann window function to reduce edge effects, which retains enough spectral weight around the topological edge to resolve the CDW order parameter. When a Fourier transform is applied, this window acts visually similar to taking the derivative in both Fourier space axes (double derivative), a common technique employed in ARPES data analysis to better visualize dispersive bands,^30,31^ while also removing spurious edge effects. These processing steps are summarized in Figure S1. True numerical derivatives in the energy axis have the effect of diminishing nondispersive features and so were avoided in this study. Figure S6 compares untreated and double derivative and Hann windowed FT-STS spectra. No symmetrization of the resultant Fourier transforms was performed. The intensity of the integrated gap LDOS for FT-STS spectra of the edge regions is artificially reduced to be visualized on the same color scale. Figure S8 shows the same data without the integrated gap LDOS, and are visualized on the same color scale using log-scale intensity. The value of the CDW order parameter was calculated by performing Gaussian fits to the constant energy cuts in Figure 4b, where we quote the mean of the fits to 1 standard deviation. All analyses were performed in Python using NumPy,^32^ SciPy,^33^ and Matplotlib.^34^

### Interacting *k*·*p* Band Structure

The electronic structure of WTe_2_ is described with a *k*·*p* model.^6,9,10^ Near the Γ point, the Hamiltonian of a four-band model reads:

1 $$ĥ\left(k\right)=\varepsilon_{+}\left(k\right)+\left[\varepsilon_{-}\left(k\right)+\delta \right]\tau^{z}+v_{x}k_{x}\tau_{x}s_{y}+v_{y}k_{y}\tau_{y}s_{0}$$

where τ^μ^ and *s* ^μ^ are Pauli matrices, representing orbital and spin degree of freedom. τ^*z*^ = ±1 refers to the d and p orbitals, respectively. The parameters used can be found in ref 6. With this set of parameters, the conduction band minimum and the valence band maximum are connected by a wavevector of length $q_{c}=\frac{1}{6}\left[2\pi /a\right]$ . The Coulomb interactions in the charge density wave phase are considered by the interacting Hamiltonian:

2 $$H_{\mathrm{int}}=\frac{1}{2N_{k}}\sum_{k,p,q,\alpha ,\beta }U\left(q\right)c_{k+q,\alpha }^{+}c_{p-q,\beta }^{+}c_{p,\beta }c_{q,\alpha }$$

where *c*_***k***,α_^+^(*c*_***k***,α_) are the creation (annihilation) operators for an electron with momentum ***k*** and orbit index α, and $U\left(q\right)=\frac{2U_{0}}{q\xi }\tanh\frac{q\xi }{2}$ is a model screened interaction with a screening length ξ = 25 nm. The value of *U*_0_ is chosen to match the gap from the self-consistent calculations to the gap observed in the experiments. To simulate the charge doping effect, we introduce a chemical potential term μ in the noninteracting part of the Hamiltonian.

### Real-Space Calculations of Excitonic Insulator Edge LDOS

The modeling of the impact of the excitonic condensate on the topological edge state was performed using a lattice-discretized version of the Bernevig–Hughes–Zhang model,^35^ with the direction of the spin–orbit coupling term taken as in the WTe_2_ model above. This choice of model is motivated by the fact that the *k*·*p* model which reproduces the low-energy band dispersion of WTe_2_ tends to greatly underestimate the penetration depth of the topological edge states (*l* < 1 nm) as compared to the experiments, where *l* ∼ 3.6 nm. The momentum space form of this Hamiltonian is

3 $$H\left(k\right)=A\left(k_{x}\tau_{x}s_{y}+k_{y}\tau_{y}s_{0}\right)+\left(M+Bk^{2}\right)\tau_{z}s_{0}+Dk^{2}\tau_{0}s_{0}$$

where *k* ^2^ = *k*_*x*_^2^ + *k*_*y*_^2^ and τ_*i*_, *s*_*i*_ are Pauli matrices representing orbital and spin degrees of freedom. The model is then discretized on a square lattice with a lattice constant *a* = 1 Å using the standard finite difference method. Calculations are performed for a ribbon of width *W* = 500 Å, which is infinite along the *x* direction (allowing the use of translational invariance), and the hard-wall boundary conditions are chosen in the *y* direction.

Comparisons are made between three different order parameters, which correspond to the results obtained through the Hartree–Fock calculations in refs 6,8 : time-reversal-preserving charge density wave (CDW), time-reversal-breaking spin density wave (SDW), and spin spiral. Each of these order parameters makes a different contribution to the real-space Hamiltonian of the system:

4 $$H_{\mathrm{CDW}}\left(r\right)=\Delta_{\mathrm{CDW}}\;\cos\left(q_{c}y\right)\tau_{0}s_{0}$$

5 $$H_{\mathrm{SDW}}\left(r\right)=\Delta_{\mathrm{SDW}}\;\sin\left(q_{c}y\right)\tau_{0}\left(s_{x}+s_{z}\right)$$

6 $$H_{\mathrm{spiral}}\left(r\right)=\Delta_{\mathrm{spiral}}\left(\cos\left(q_{c}y\right)\tau_{0}s_{x}+\sin\left(q_{c}y\right)\tau_{0}s_{z}\right)$$

where *q*_c_ = 0.26 Å^–1^ is the momentum separation between electron and hole pockets as found from the experimental determination of the band dispersion. The modulation direction is perpendicular to the edge; therefore, it does not affect translational invariance along the *x* direction. Local density of states is then calculated at energy *E* = 3.5 meV for each of the order parameters separately and plotted in the vicinity of the edge of the ribbon, resulting in the data presented in Figure 3. In performing the calculations, KWANT package was used.^36^ The parameter values are *A* = 0.25 eV Å, *B* = −10 eV Å^2^, *D* = −8 eV Å^2^, *M* = −5 meV, Δ_CDW_ = 5 meV, Δ_SDW_ = 22 meV, and Δ_spiral_ = 20 meV. The model parameters are chosen such that the penetration depth of the topological edge state is about 36 Å, when no order parameter is present, which is the penetration depth seen in the experiment.

## Supplementary Material



## Notes

Additional FT-STS data processing method details, figures of alternate data processing, and comparison between data acquired with and without out-of-plane magnetic field (*B*_⊥_ = 3 T) (PDF)
