# Supplementary material for: Observation of the Charge Density Wave Excitonic Order Parameter in Topological Insulator Monolayer WTe2
Source: ACS Nano. 2025 Sep 4;19(36):32374–81. doi: 10.1021/acsnano.5c08005 (PMC12445006; doi:10.1021/acsnano.5c08005)
Supplement: Supplementary file 1 [file nn5c08005_si_001.pdf]

Supporting Information

Observation of charge density wave excitonic order parameter in topological insulator monolayer WTe_2

Liam Watson[1,2], Joan Ripoll[3,5], Zhengjue Tong[6], Amit Kumar[6], Yande Que[6], Yang-Hao Chan[7], Hsin Lin[8], Shantanu Mukherjee[9], Manuela Garnica[5,10], Mark T Edmonds[1,2], Michał Papaj[11,12], Amadeo L Vazquez de Parga[3,4,5], Bent Weber[*,2,6], Iolanda Di Bernardo[*,1,2,3,4], and Michael S Fuhrer[*,1,2]

[1] *School of Physics and Astronomy, Monash University, Clayton, 3800, VIC, Australia*
[2] *Australian Research Council Centre of Excellence in Future Low-Energy Electronics Technologies, Monash University, Clayton, 3800, VIC, Australia*
[3] *Departamento de Física de la Materia Condensada, Universidad Autónoma de Madrid, Madrid, 28049 Spain*
[4] *IFIMAC Condensed Matter Physics Center, Madrid, 28049, Spain*
[5] *Instituto Madrileño de Estudios Avanzados en Nanociencia (IMDEA-Nanociencia), Madrid, 28049 Spain*
[6] *School of Physical and Mathematical Sciences, Nanyang Technological University, Singapore, 637371 Singapore*
[7] *Institute of Atomic and Molecular Sciences, Academia Sinica, Taipei 106319, Taiwan*
[8] *Institute of Physics, Academia Sinica, Taipei 155201, Taiwan*
[9] *Quantum Centre for Diamond and Emergent Materials, Indian Institute of Technology Madras, Chennai, Tamil Nadu 600036, India*
[10] *Instituto Nicolás Cabrera, Universidad Autónoma de Madrid, 28049 Madrid, Spain*
[11] *Department of Physics, University of Houston, Houston, Texas 77204, USA*
[*] *E-mail: b.weber@ntu.edu.sg; iolanda.dibernardo@monash.edu; michael.fuhrer@monash.edu*

Fourier transform scanning tunnelling spectroscopy data processing

All Fourier transform scanning tunnelling spectroscopy (FT-STS) data in the main text are processed with the Hann (cosine) window [1, 2], defined by:

$$\frac{1}{2}\left[1-\cos\left(\frac{2\pi n}{N}\right)\right]$$

where N is the number of STS spectra acquired along the line of acquisition (*e.g.* Figure 2d in the main text). This window acts visually similar to taking the derivative in both Fourier space axes (double derivative), a common technique employed in ARPES data analysis to better visualise dispersive bands [2, 3]. A summary of the data processing steps performed to FT-STS data are shown in Figure S1.

Numerical derivatives of the real space data are calculated with the Savitzky-Golay filter [4] from the Scipy package [5]. Double derivatives of FT-STS data were produced by the fast-Fourier transform of two linear fittings using the Savitzky-Golay method applied over windows of $\sim$ 18 mV in energy and $\sim$ 4 Å in real space. These figures are provided below as comparisons with Hann window processing in Figure S6.

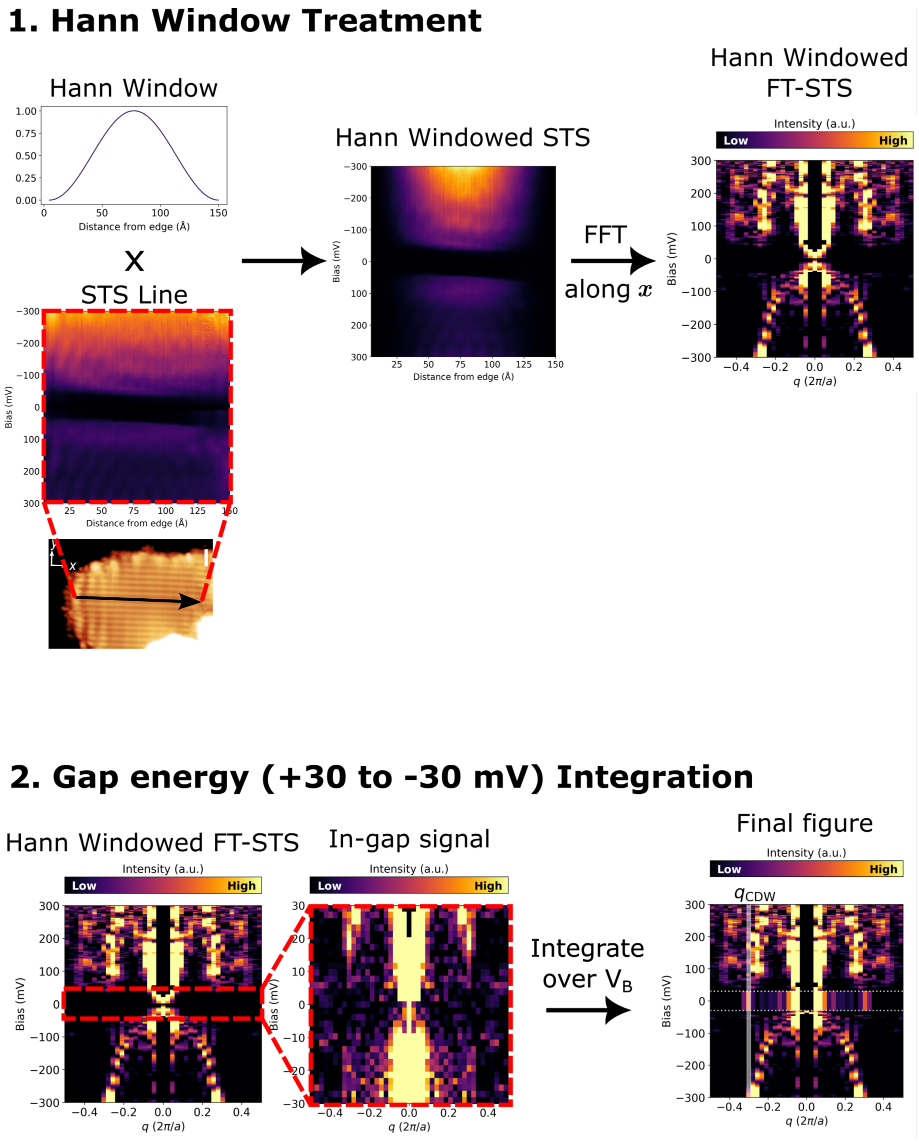

Figure S1: A summary of the data processing steps applied to FT-STS data.

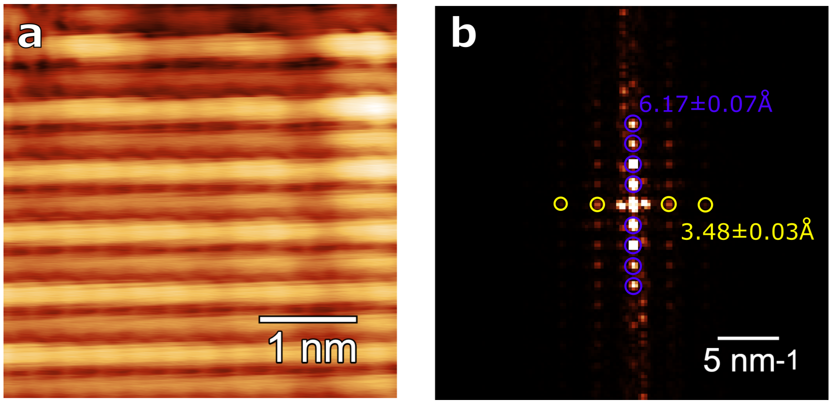

Figure S2: Determination of lattice parameters. **a.** Atomically resolved topograph of the WTe_2 surface grown on HOPG. **b.** Corresponding FFT of a. Additional topographs from different regions were used to calculate the mean and standard deviation of the lattice constants.

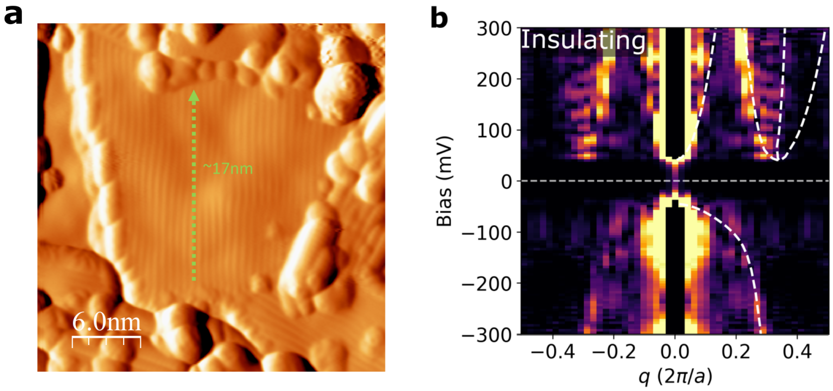

Figure S3: **a.** Topography (derivative) of monolayer WTe_2 grown on graphene over SiO_2/Si. **b.** FFT-STS of the 2D bulk region indicated by the green arrow in a (length = 16.6 nm), displaying insulating behaviour at gate voltage $V_G = 10$ V.

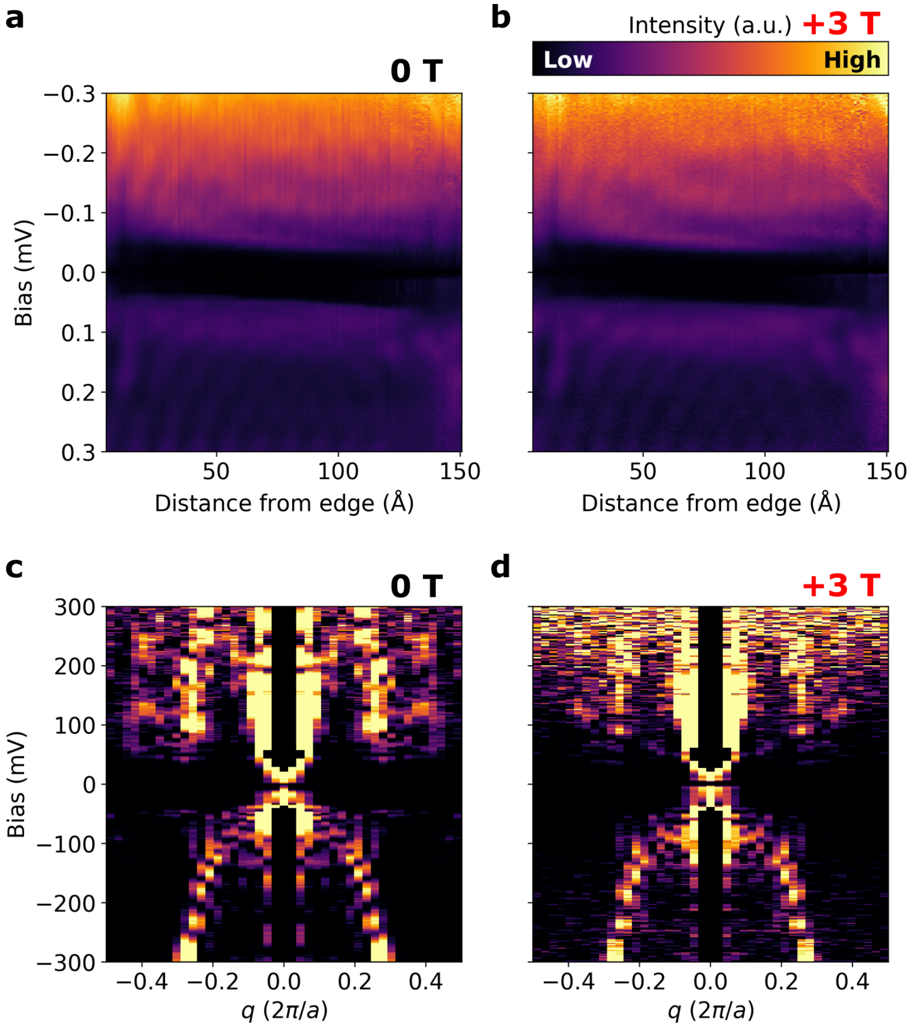

Figure S4: A comparison of the **a,b.** STS and **c,d.** FT-STS spectra for the edge region of WTe_2 in **a,c.** the absence of a magnetic field, and **b,d.** the presence of an out-out-plane magnetic field $B_\perp$ of strength $+3$ T.

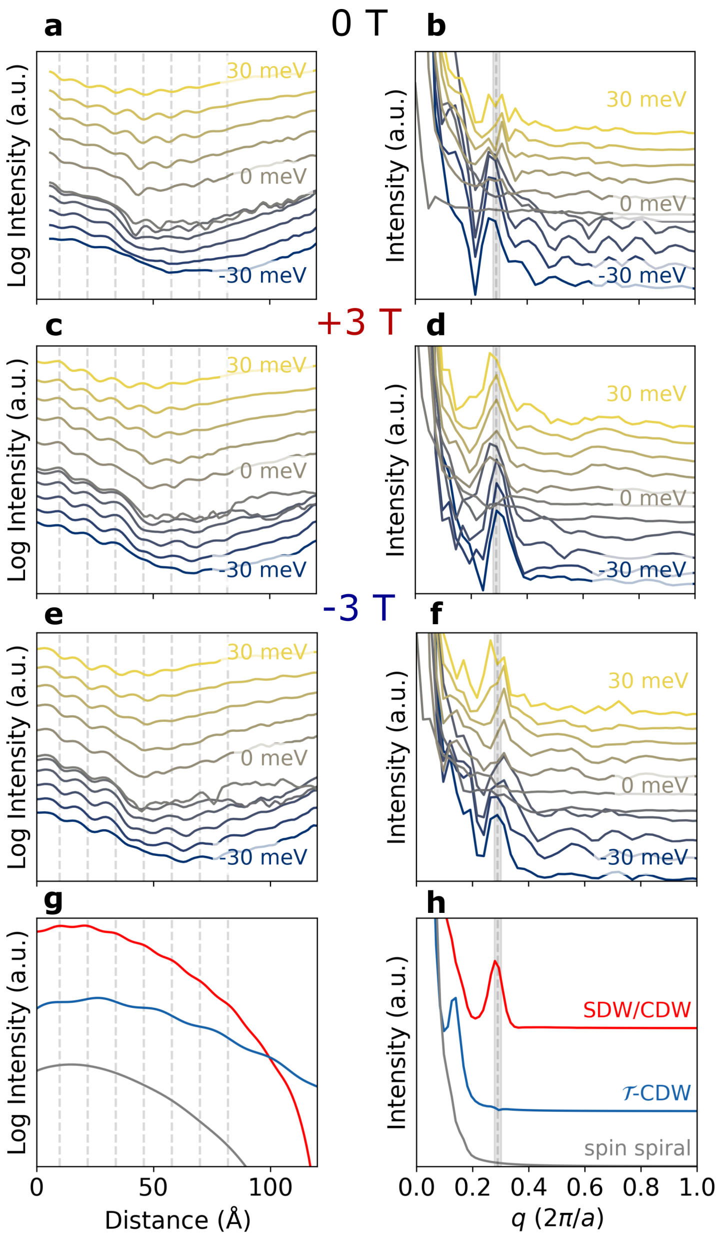

Figure S5: A comparison of the modulations to the LDOS inside the gap energies (+30 to −30 mV) in **a,b.** no field, and in the presence of an out-out-plane magnetic field $B_\perp$ of strength **c,d.** +3 T and **e,f.** −3 T. **a,c,e.** Constant energy cuts of the LDOS inside the insulating gap as a function of distance away from the edge, and **b,d,f.** their Fourier transforms, respectively. **g.** Calculated topological edge LDOS, and **h.** Fourier transform for the three different excitonic phases: SDW/CDW (red), $\mathcal{T}$-CDW (blue), and spin spiral (grey), offset for clarity.

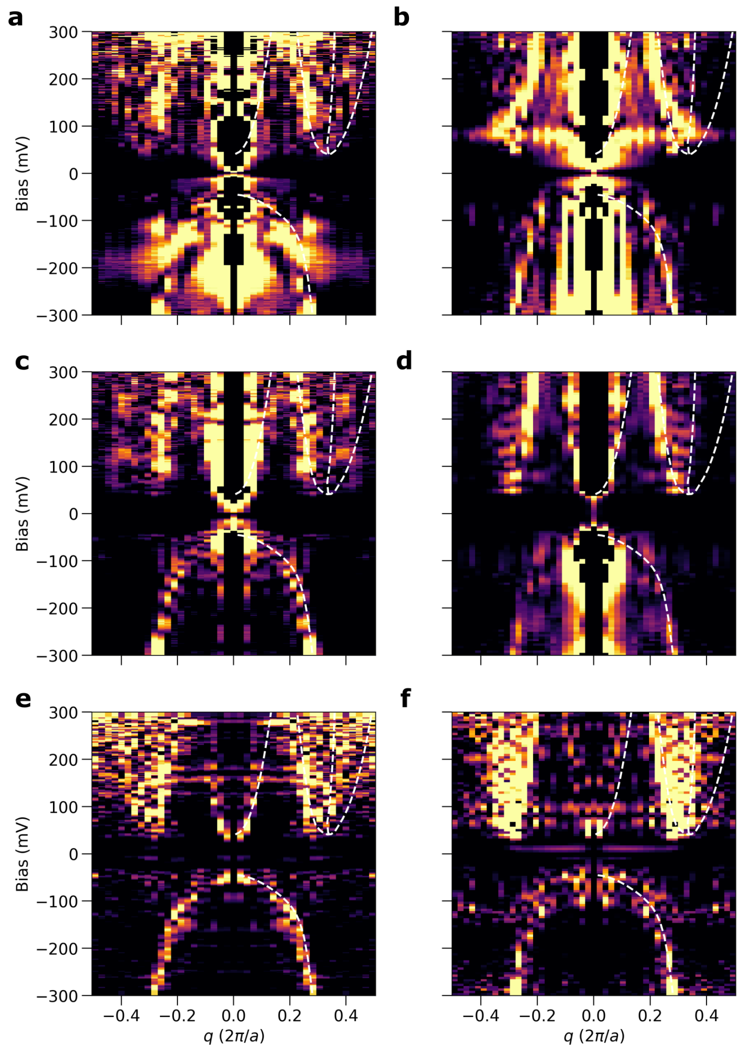

Figure S6: A comparison of data processing techniques for FT-STS spectra for **a,c,e.** edge, and **b,d,f.** 2D bulk regions of WTe_2. **a,b.** No processing, **c,d.** Hann window, and **e,f.** double derivative. The calculated scattering vectors from the interacting $k \cdot p$ band structure are indicated with white dashed lines.

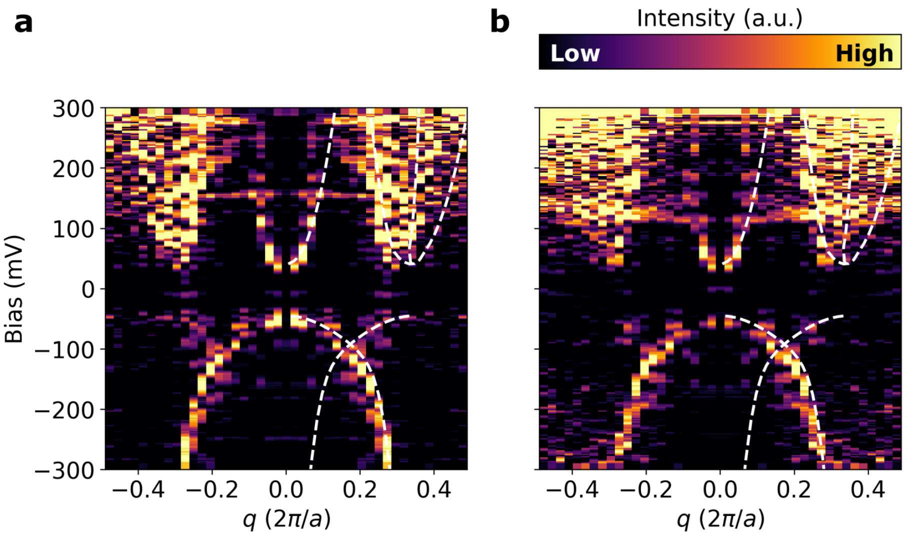

Figure S7: Double derivative processing of the FT-STS spectra of the WTe_2 edge region with $B_\perp$ field **a.** 0 T, and **b.** +3 T. The scattering of the folded hole pocket is indicated.

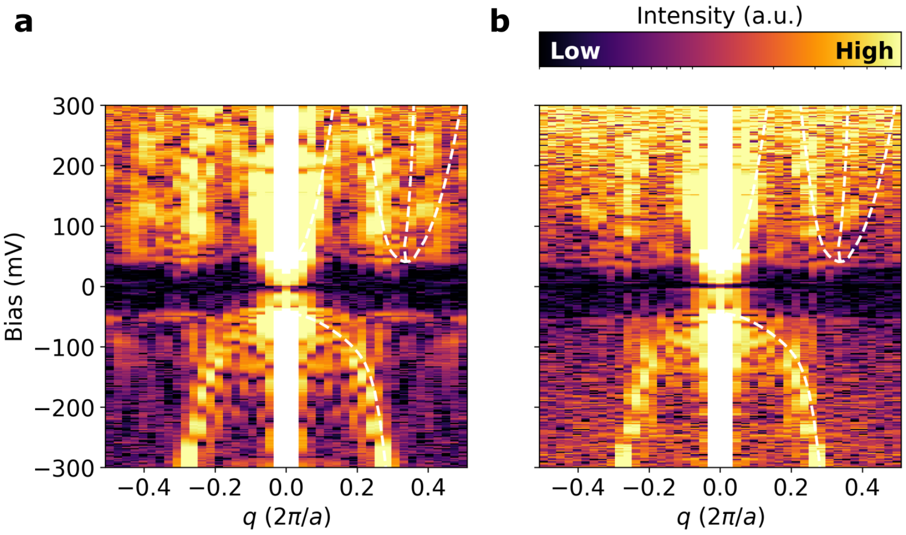

Figure S8: FT-STS spectra of the WTe_2 edge region with log-scale intensity and $B_\perp$ field **a.** 0 T, and **b.** +3 T.

References

(1) Harris, C. R. et al. *Nature* **2020**, *585*, 357–362.

(2) Harris, F. J. *IEEE Proceedings* **1978**, *66*, 51–83.

(3) Li, R.; Zhang, X.; Miao, L.; Stewart, L.; Kotta, E.; Qian, D.; Kaznatcheev, K.; Sadowski, J. T.; Vescovo, E.; Alharbi, A.; Wu, T.; Taniguchi, T.; Watanabe, K.; Shahrjerdi, D.; Wray, L. A. *Journal of Electron Spectroscopy and Related Phenomena* **2020**, *238*, 146852.

(4) Savitzky, A.; Golay, M. J. E. *Analytical Chemistry* **1964**, *36*, 1627–1639.

(5) Virtanen, P. et al. *Nature Methods* **2020**, *17*, 261–272.
